# Metformin Improves Biochemical and Pathophysiological Changes in Hepatocellular Carcinoma with Pre-Existed Diabetes Mellitus Rats

**DOI:** 10.3390/pathogens10010059

**Published:** 2021-01-11

**Authors:** Maysa A. Mobasher, Mousa O. Germoush, Hala Galal El-Tantawi, Karim Samy El-Said

**Affiliations:** 1 Department of Pathology, Biochemistry Division, College of Medicine, Jouf University, Sakaka 41412, Saudi Arabia; 2 Department of Clinical Pathology, El Ahrar Educational Hospital, Ministry of Health, Zagazig 44511, Egypt; 3 Biology Department, College of Science, Jouf University, Sakaka 41412, Saudi Arabia; mogermoush@ju.edu.sa; 4 Zoology Department, Faculty of Science, Ain Shams University, Cairo 11566, Egypt; halatantawy@sci.asu.edu.eg; 5 Chemistry Department, Biochemistry Division, Faculty of Science, Tanta University, Tanta 31527, Egypt; kareem.ali@science.tanta.edu.eg

**Keywords:** diabetes mellitus, streptozotocin, hepatocellular carcinoma, diethylnitrosamine, metformin

## Abstract

Hepatocellular carcinoma (HCC) is one of the world’s most widely recognized malignant tumors that accounts for 90% of all the primary liver cancers and is a major cause of death from cancer, representing half a million deaths per year. Obesity and associated metabolic irregularities, particularly diabetes mellitus (DM) and insulin resistance, are important risk factors for the advancement of HCC. Recently, retrospective studies showed that metformin (MET) could protect the hepatic tissues in pre-existing diabetes mellitus from HCC. The purpose of this study was to assess the role of MET treatment in the pre-existing diabetic rats before and after HCC induction by diethylnitrosamine (DEN). Thirty-five male Sprague Dawley albino rats were partitioned into the following groups: Group 1 (Gp1) was the control. Gp2 was injected intraperitoneally (i.p) with streptozotocin (STZ) (80 mg/kg) and DEN (50 mg/kg/7 weeks). Gp3, Gp4, and Gp5 were injected as in Gp2 and treated with MET (150 mg/kg) before and/or after HCC induction. Biochemical parameters including liver functions, lipid profile, and oxidative stress biomarkers were determined. Furthermore, histological and immunohistochemical changes were assessed in all groups. Our results illustrated that the group of rats that were treated with STZ and DEN had significant changes in both liver functions and were associated with alterations in the liver histopathological architectures. Treatment with MET before or after HCC induction ameliorated the cellular changes in the liver tissues; however, the utmost protection was found in a group of rats, which were treated with MET before and after HCC induction.

## 1. Introduction

Uncontrolled diabetes mellitus (DM) leads to several complications that might be risk factors for the incidence of hepatocellular carcinoma (HCC) [1,2]. HCC is a widespread kind of primary liver malignancy which has been considered the world’s main cause of cancer-related death [3]. In fact, HCC was found more frequently in individuals with low socioeconomic status due to improper access to health care [4]. In developing countries, including Egypt, there is a high incidence of HCC [5]. Development of HCC depends on upregulated signals from the insulin-like growth factors (IGFs) [6]. Insulin-dependent pathway dysregulation has been identified as a risk factor for HCC [7]. The relationship between diabetes and HCC has been assessed in huge populaces; emerging from this, preclinical investigations have demonstrated that anti-diabetic medications may alter the risk of developing HCC [8,9,10,11]. The anti-diabetic medication metformin (MET), which diminishes insulin resistance, has been reported to play an important role against the incidence of cancer [12].

Retrospective studies showed that DM individuals receiving MET had a low risk of developing cancer, while patients receiving sulphonylureas and insulin had an expanded cancer frequency and mortality rates [13]. Moreover, these consequences highlighted that the protective impact of MET depended mainly on its dose [14]; moreover, MET has been shown to have antioxidant, antiaging, and antitumor activities in both vitro and vivo, which reduce the risk of several solid tumors, such as pancreatic, breast, colorectal, and prostate cancers [15,16,17,18].

Reactive oxygen species (ROS) and their derivatives induce oxidative DNA damage and abnormal protein expression, which could help in the development of various diseases [19]. During DM, exaggerated ROS production damages the lipids, proteins, and genetic materials via triggering of various signaling cascades, alteration of gene expression that control cellular proliferation, angiogenesis, and metastasis [20]. Subsequently, ROS immediate pathophysiological changes prompt the pre-neoplastic initiatory cells’ growth.

The preventive impact of MET on HCC development in DM patients and the direct anti-HCC effect of MET have been reported [21,22]. The mechanism of MET action in DM patients on HCC prevention and treatment is supposed to be related to the AMPK pathway, whereas MET activates AMPK expression by increasing the cellular energy stress, which triggers insulin/IGF-1 signaling inhibition that is involved in the control of cancer glycolysis and carcinogenesis [23]. Additionally, MET contributes to an increase in the glutathione (GSH) content, which protects against oxidative stress. MET promotes the increase of NAD/NADH ratio, thus increasing the expression of NAD-dependent protein deacetylase sirtuin-1 (SIRT1) [24]. The target of this study was to investigate the role of MET treatment under a distinctive setting in the pre-existing diabetic rats induced for HCC.

## 2. Materials and Methods

### 2.1. Chemicals

Diethylnitrosamine (DEN) and streptozotocin (STZ) were purchased from Sigma (St. Louis, MO, USA). Metformin (MET) was purchased from a local pharmacy in Saudi Arabia. All biochemical kits were purchased from the Bio-Diagnostic company (Cairo, Egypt). The primary and secondary antibodies for immunohistochemical investigations were purchased from Dako (Glostrup, Denmark).

### 2.2. Experimental Design

Thirty-five male Sprague Dawley albino rats (100 ± 5 g) were received from the National Research Center (NRC, Cairo, Egypt) and housed randomly at seven rats per cage in 12 h/12 h dark/light cycles under standard temperature and humidity laboratory conditions. Animals were carefully observed daily, and their body weights were recorded, while food and water intakes were accurately measured each week to assess any signs of toxicity or abnormality throughout the experiment.

### 2.3. DM and HCC Inductions

**Figure 1:**
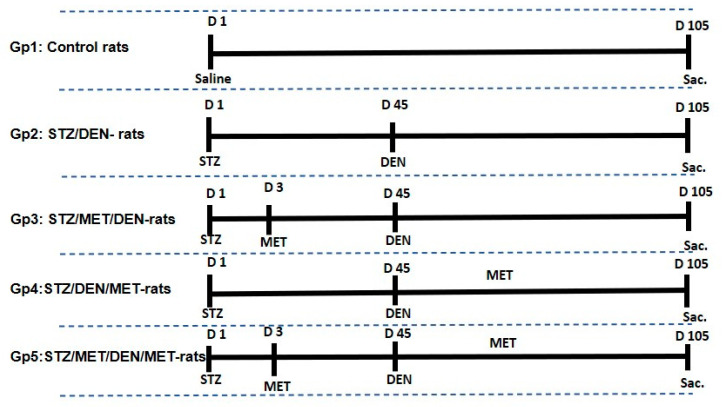
Experimental design showing the different groups under the study with time course injections of streptozotocin (STZ), diethylnitrosamine (DEN) and metformin (MET). Gp1: received only saline. Gp2 (STZ/DEN): injected intraperitoneally (i.p.) with a single dose of STZ (80 mg/kg) and after 45 days were injected i.p with DEN (50 mg/kg). Gp3 (STZ/MET/DEN): injected i.p with STZ, 3 days later rats were treated by gavage with 150 mg/kg of MET each other day for 102 days, then at day 45 rats were injected with DEN once weekly. Gp4 (STZ/DEN/MET): injected i.p with STZ, 45 days later DEN was injected once per week and rats were treated by gavage with 150 mg/kg of MET. Gp5 (STZ/MET/DEN/MET): injected i.p. with STZ, 3 days later injected with MET, 45 days later both DEN and MET treatment started.

For diabetes induction, rats were intraperitoneally (i.p.) injected with a single dose of STZ (80 mg/kg) [25]. For HCC induction, rats were i.p. injected with DEN (50 mg/kg) (Sigma, St. Louis, MO, USA) once weekly for seven weeks [26]. For MET treatment, rats were treated by gavage with 150 mg/kg each other day for 102 days. To establish the diabetic/HCC in rats, STZ was injected, as mentioned above, and after 45 days, DEN was injected (Figure 1).

### 2.4. Experimental Animals

Rats were divided into five groups (*n* = 7) as the following: Gp1 was served as the negative control. Gp2 was injected with STZ/DEN, Gp3 was injected with STZ/MET/DEN, Gp4 was injected with STZ/DEN/MET, and Gp5 was injected with STZ/MET/DEN/MET. At the last day of the experiment, day 105, all rats were sacrificed under ethyl ether anesthesia, and cadavers were burned in animal incinerators under the supervision of the Faculty of Science, Tanta University. Gross examinations were performed macroscopically for all groups during sacrifice. Blood samples were collected from all groups from arterial blood vessels and heart chambers for hematological investigations, and sera were separated by centrifugation for biochemical analysis. Liver tissues were collected and liver homogenates were prepared in ice-cold phosphate buffer saline (PBS). The resulting supernatants were used for biochemical analysis. Furthermore, liver tissues were separated and fixed in buffered formalin for histological and immunohistochemical investigations.

### 2.5. Haematological and Biochemical Profiling

Hemoglobin (Hb) levels, hematocrit (Hct %), platelet count, total counts of red blood cells (RBCs), white blood cells (WBCs), and differential leucocyte count were determined by the usage of auto hematology analyzer (BC-3200, Mindray, Guangdong, China). Serum aspartate aminotransferase (AST) and alanine aminotransferase (ALT) were detected as described [27]. Arginase activities were detected by using Bio-Diagnostics kits [28]. Hepatic alkaline phosphatase (ALP) and total protein (TP) were assessed as previously described [29,30], respectively. Albumin and total bilirubin (TB) were assessed using kits following the manufacturer’s instructions as previously described [31,32]. Urea and creatinine levels were measured using kits as described [33,34].

Serum cholesterol, triglycerides, and HDL-cholesterol were determined using a quantitative kit based on the previously described methods [35,36,37], respectively. Low-density lipoprotein cholesterol (LDL) was calculated consistent with Friedewald et al. [38] as follows: LDL = Total cholesterol—HDL—(Triglycerides/5). Superoxide dismutase (SOD) activity was determined, in accordance with Paoletti and Mocali (1990) [39]. Catalase activity was determined according to Aebi (1984) [40]. Reduced glutathione (GSH) determination was based on the method of Paglia and Valentine (1967) [41]. Finally, Malondialdehyde (MDA) levels were determined according to the strategy adopted by Li and Chow (1994) [42].

### 2.6. Histopathological Investigations

The tissue of the liver was processed for the light microscopic examination and fixed in 10% formalin. Paraffin blocks were prepared after rinsing in different grades of alcohol and xylene. Sectioning of the paraffin blocks into sections (5 μm) and holding on glass slides for staining with hematoxylin and eosin stain. Examination of the stained tissues was conducted and photographed with a light microscope (Optica light microscope (B-350)) to examine gross cellular damage [43].

### 2.7. Immunohistochemical Staining for PCNA and Caspase-3 Detection

The liver tissues were fixed in 10% buffered formalin for 18–24 h and transferred to 70% ethanol overnight. Tissues were processed in temperature less than 60 °C. The tissue sections were drained, and excess antibodies were wiped. Incubation with the primary antibody was carried out, and proliferating cell nuclear antibodies (PCNA) (19A2) at a dilution of 1:400 for 30 min at RT and the rabbit anti-cleaved caspase antibodies were processed on deparaffinized tissue sections using Vectastain Elite avidin-biotin-immunoperoxidase kit (Dako, Glostrup, Denmark). From each of the same tissues, the negative control slides were incubated with control antibody (normal nonimmunized mouse immunoglobulin in diluent) for 30 min. Washing of the slides was done in two changes of 1× Automation Buffer, 5 min each. Counterstaining of the tissue sections was carried out with Mayer’s hematoxylin (Sigma). Negative controls were obtained by leaping the application of the primary antibody.

### 2.8. Statistical Analysis

Data were presented as mean ± SD. One-way analysis of variance (ANOVA) was used to determine whether there were any statistically significant differences between the means of different groups. If there was a significant difference between means, Tukey’s method for multiple comparisons was used to detect all pairwise differences between group means to determine specifically which means are different. For all statistical tests, *p* < 0.05 was considered to be statistically significant. Data and statistical analysis were performed using Excel 2016, and Minitab version 19 (LLC, State College, PA, USA). In all tables and figures means that do not share a letter are significantly different.

## 3. Results

### 3.1. Injection with STZ, DEN, and MET Treatment Decreased Rats’ Body Weights

**Figure 2:**
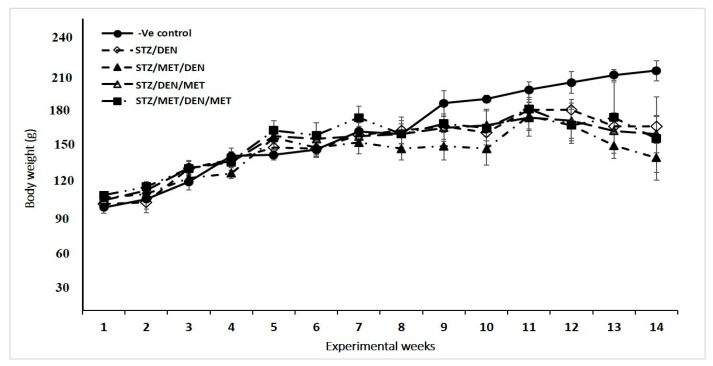
Body weight changes over 14 weeks of treatments with STZ, DEN, MET, or their combinations, according to the experimental design.

The results showed that STZ and DEN injections decreased the total body weight of rats starting from week 9. The treatment with MET, however, before and/or after induction of the pre-existing diabetic rats with DEN was not able to return the body weights to normal levels until week 14. The groups of rats treated with MET post-STZ injection and before DEN induction (Gp3) indicated the most extreme decrease in the all-out body weight when compared with other groups (Figure 2).

### 3.2. Injection with STZ and DEN Increased the Number of RBCs and WBCs

The total number of leukocytes (WBCs) increased significantly in the group of rats injected with STZ and DEN (Gp2) (Table 1). The number of WBCs also increased in the group of diabetic rats which was treated with MET after DEN injection (Gp4) and in the diabetic rats treated with MET before and after induction with DEN (Gp5) (Table 1). The percentage of monocytes was increased only in Gp5 when compared to control groups (data not shown).

### 3.3. Effect of MET/DEN on the Liver Functions, Lipid Profile, and the Antioxidant Biomarkers

**Figure 3:**
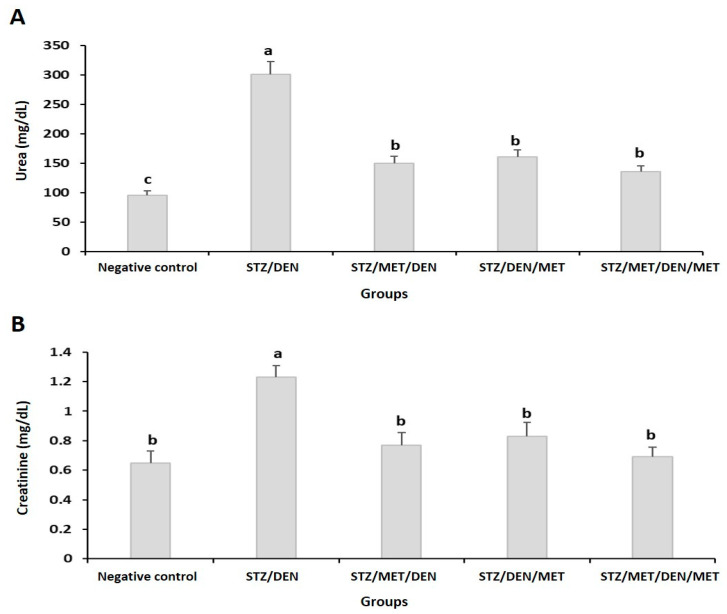
Levels of the urea (**A**) and creatinine (**B**) in the different groups under study. The results are means of seven rats per group under different treatment conditions as indicated in the experimental design section. Bars represent standard deviation. Columns with different lower-case letters indicate a significant difference between all the studied groups at *p* < 0.05 (Tukey’s test).

**Figure 4:**
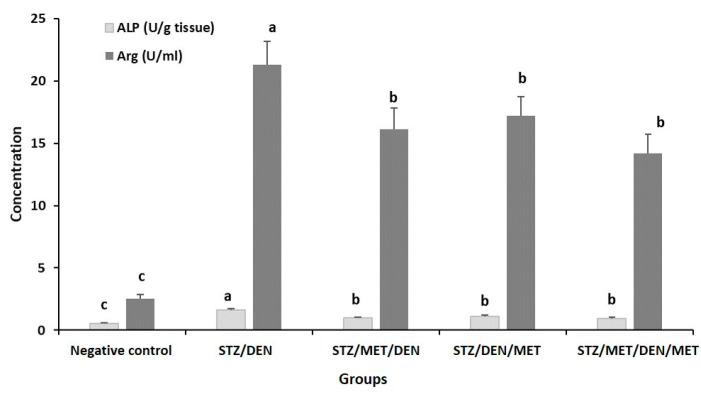
Levels of alkaline phosphatase (ALP) and arginase (Arg) activities in the sera of the different groups under study. The results are means of seven rats per group under different treatment conditions as indicated in the experimental design section. Bars represent standard deviation. Columns with different lower-case letters indicate a significant difference between all the studied groups at *p* < 0.05 (Tukey’s test).

**Figure 5:**
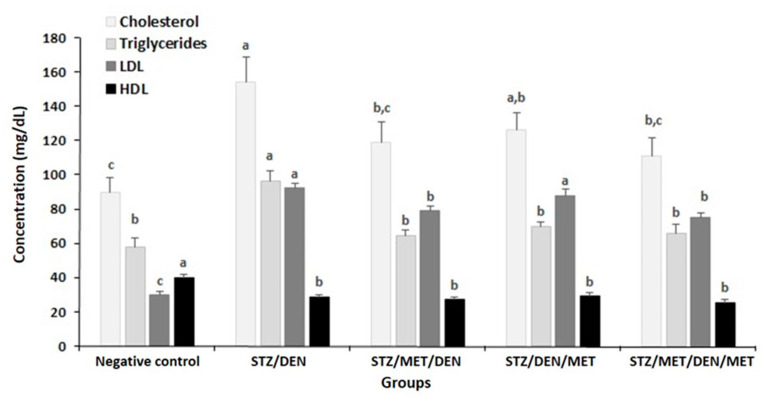
The concentrations of cholesterol, triglycerides, low-density lipoprotein cholesterol (LDL), and high-density lipoprotein cholesterol (HDL) in the sera of the different groups under study. The results are means of seven rats per group under different treatment conditions as indicated in the experimental design section. Bars represent standard deviation. Columns with different lower-case letters indicate a significant difference between all the studied groups at *p* < 0.05 (Tukey’s test).

The results showed that diabetic rats injected with DEN (Gp2) to establish HCC had a significant increase in the levels of ALT, AST, TB (Table 2), urea, creatinine, ALP, and arginase levels when compared to control (Figure 3 and Figure 4). Furthermore, the levels of cholesterol, triglycerides, and LDL also increased in this group when compared to their control (Figure 5). The diabetic rats treated with MET before HCC induction (Gp3) decreased all these biochemical changes mentioned above (Table 2, Figure 3, Figure 4 and Figure 5). The treatment of diabetic rats with MET before and after DEN induction (Gp5) showed the maximum improvement as they revealed the lowest levels of ALT, AST, urea, creatinine, ALP, and arginase when compared to other groups (Table 2, Figure 3, Figure 4 and Figure 5).

On the contrary, the results in Gp2 showed that concomitant with the increase of the previous parameters, a significant reduction in the TP levels, albumin, and HDL was seen when compared to the control (Table 2, Figure 5).

**Figure 6:**
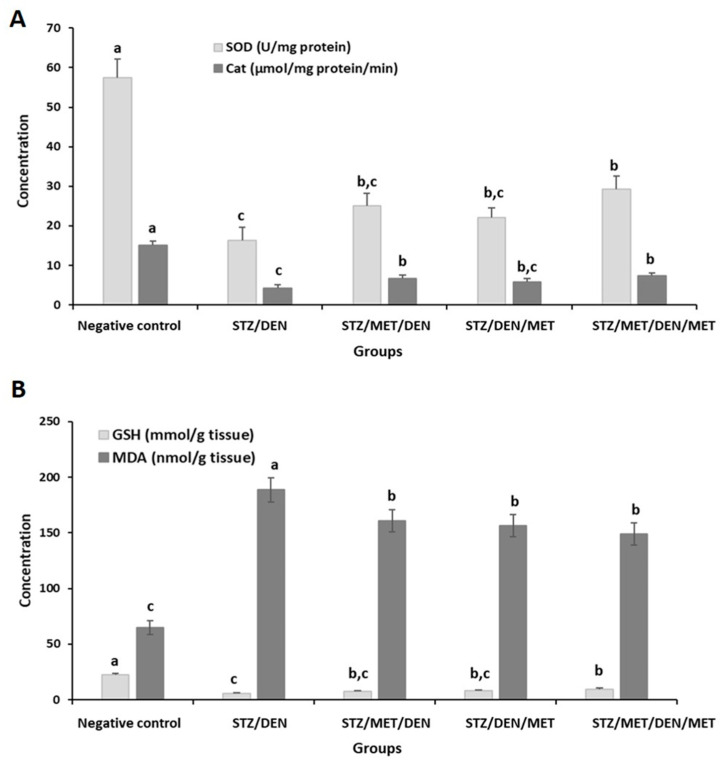
The activities of superoxide dismutase (SOD), catalase (CAT) (**A**), and the concentrations of glutathione (GSH) and malondialdehyde (MDA) (**B**) in the liver tissues of the different groups under study. The results are means of seven rats per group under different treatment conditions as indicated in the experimental design section. Bars represent standard deviation. Columns with different lower-case letters indicate a significant difference between all the studied groups at *p* < 0.05 (Tukey’s test).

Furthermore, in this group, there was an elevation in MDA levels that was associated with decreases in SOD, CAT, and GSH levels. Treatment with MET before or after induction of HCC increased the previously ameliorated oxidative stress parameters; however, the maximum improvement was found in the group of rats treated with MET before and after HCC induction (Gp5) which showed the lowest increase in MDA levels and the lowest decrease in SOD, CAT, and GSH levels, when compared to other groups (Figure 6).

### 3.4. Histological and Immunohistochemical Investigations of Liver Tissues

**Figure 7:**
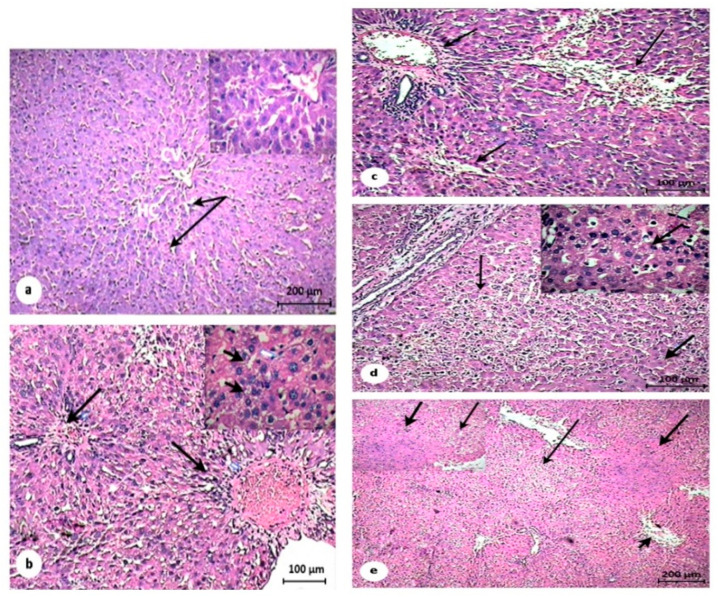
Photomicrographs showing liver sections of rats stained by hematoxylin and eosin. (**a**) Control group (G1) revealing the hepatic strands (arrows) of the hepatic cells (HC) arising from the central vein (CV), that was lined by the normal endothelial cells (arrowheads). (**b**) STZ/DEN treated group (G2) showing primary liver cirrhosis and the portal veins markedly enlarged within marginal loose connective tissues. The population of small lymphocytes and inflammatory cells are present (long arrows). The hepatocytes have also eroded, as exhibited by the piecemeal necrosis of some hepatocytes (thin arrows). (**c**) STZ/MET/DEN treated group (G3) showing cellular infiltration at the portal tracts (thin arrows) and apparent marginal loose connective tissues at the central vein (thick arrow). Most of the hepatocytes normally appear with wide blood sinusoids. (**d**) STZ/DEN/MET treated group (G4) showing severe necrosis of hepatocytes with marked pyknotic nuclei (thin arrows) at certain sites, while other sites demonstrate more or less normal hepatocytes with slight cytoplasmic vacuolation and vesicular nuclei (thick arrows), but the tissue does not show hepatocellular carcinoma. (**e**) STZ/MET/DEN/MET treated group (G5) showing the hepatic lobulation. Focal necrotic (thin arrows) and some apoptotic cells (thick arrows). Additionally, the reduction of the cellular infiltration in the hepatic parenchyma and portal veins (arrowheads) was observed.

The control untreated tissue sections revealed the liver tissue, which consists of many anastomosing strands of hepatocytes. The hepatocytes appeared as polyhedral cells with acidophilic cytoplasm and rounded central vesicular nuclei. The blood sinusoids showed interlobular spaces surrounding the central vein (Figure 7a). Treatment with STZ for 45 days followed by DEN for 60 days increased the inflammation of the hepatic tissue and resulted in induction of the primary liver cirrhosis. The portal veins markedly enlarged and were surrounded by peripheral loose connective tissues. Additionally, many populations of small lymphocytes and inflammatory cells appeared; the hepatocytes eroded, as exhibited by the piecemeal necrosis of some hepatocytes (Figure 7b). The apparent disappearance of the hepatic cirrhosis revealed in all STZ/MET/DEN, STZ/DEN/MET, and STZ/MET/DEN/MET treated groups (Gp3, Gp4, and Gp5, respectively). STZ/MET/DEN treated group showed cellular infiltration at the portal tracts, marked appearance of marginal loose connective tissues at the central veins, and disappearance of the malignant cells. In STZ/DEN/MET treated animal group (Gp4) showed apparent severe necrosis of hepatocytes with marked pyknotic nuclei at particular sites, while many sites demonstrated more or less normal hepatocytes with profound degenerative changes (Figure 7c,d). Hepatic tissue regained more or less the normal architecture of hepatocytes and lobules in STZ/MET/DEN/MET treated group (Gp5), in which it was recorded that the hepatic lobulation exhibited significant improvement accompanied with mild infiltration at the portal veins (Figure 7e).

**Figure 8:**
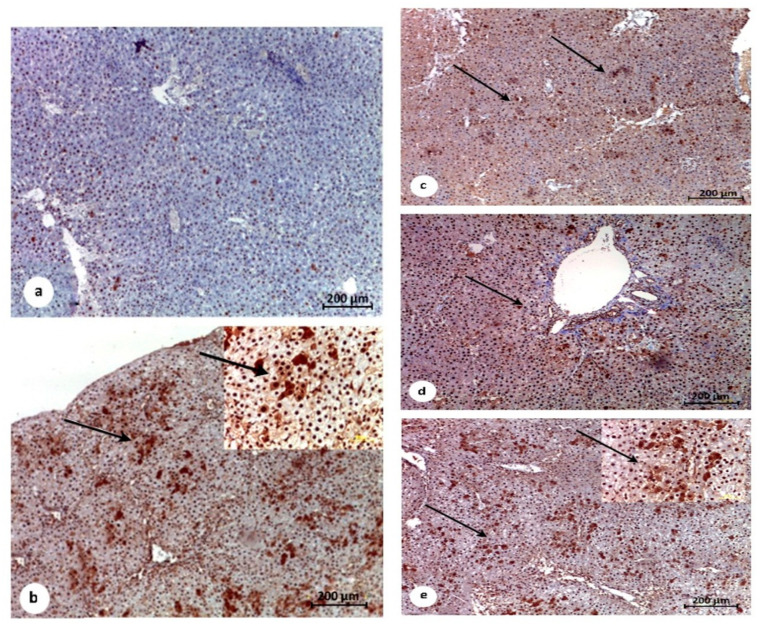
Immunohistochemical photomicrographs of liver tissues for demonstrations of proliferating cell nuclear antigen (PCNA). Counterstained blue nuclei of the hepatocytes are negatively expressed for PCNA in the control untreated group (G1) (**a**) STZ/DEN and STZ/MET/DEN treated groups G2 and G3 showing significant expression of PCNA immunolabelling cells (thin arrows) (**b**,**c**). Remarkable improvement and less expression of PCNA immunolabelling cells are showing in both STZ/MET/DEN/MET and STZ/ DEN/MET treated groups G5 and G4 respectively (thin arrows) (**d**,**e**).

Liver sections of the control untreated group (Gp1) revealed normal hepatic architecture with negative proliferating cell nuclear antibodies (PCNA) as an immunolabelling marker (Figure 8a). Treated group STZ/DEN (Gp2) in which the experimental animals were treated (with DEN and MET) revealed a high expression of PCNA at the pericentral area of the hepatic tissue (Figure 8b). Pretreatment with MET in STZ/MET/DEN treated group (Gp3) and in STZ/DEN/MET treated group (Gp4) revealed slight positive expression of PCNA in the hepatic tissues (Figure 8c,d), while STZ/MET/DEN/MET treated group (Gp5) revealed weak expression for PCNA (Figure 8e).

**Figure 9:**
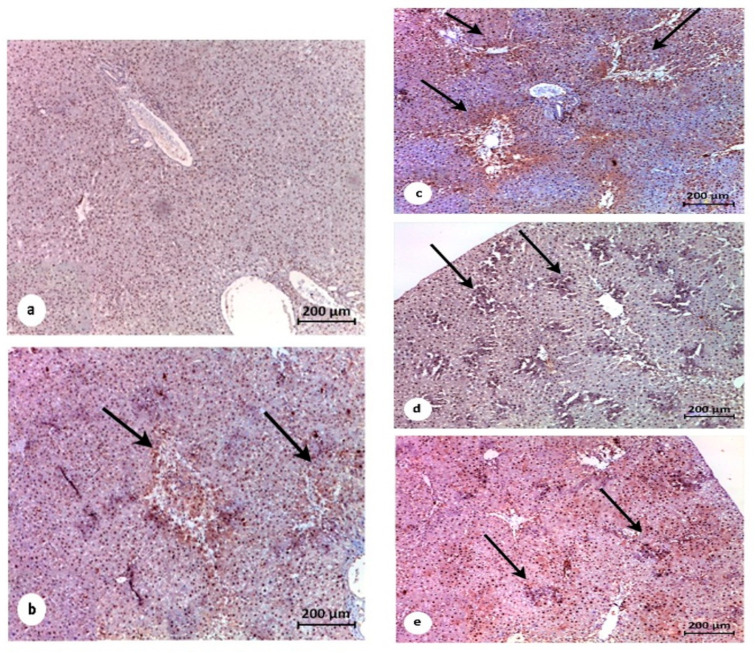
Immunohistochemical photomicrograph of liver tissues for demonstration of apoptotic cell population marker caspase-3. Counterstained nuclei of the hepatocytes are negatively expressed for caspase in the control untreated group (G1) (**a**). Significantly high expression of the apoptotic marker observed in STZ/DEN treated group (G2). Notably, positive immunolabelling for caspase-3 is showing at the surrounding of the portal veins (thin arrows) in both STZ/DEN and STZ/MET/DEN treated groups (G2 and G3) (**b**,**c**). Moderate significance of caspase-3 marker is showing in STZ/DEN/MET treated group (G4) (**d**). STZ/MET/DEN/MET treated group (G5) showing decline in the immunolabelling cells (**e**).

The activity of caspase-3 marker for apoptotic cells was examined as an immunolabelling marker for the hepatic tissues. The non-treated control group (Gp1) showed negative expression (Figure 9a). Treated group Gp4 showed reduced expression of caspase-3 activity in the hepatic tissues as many scattered patches were noticed (Figure 9d) while treated groups Gp3 and Gp5 demonstrated apparent improvement and reduction of the immunoexpressed cells with caspase-3 antibodies marker (Figure 9c,e) that appeared concentrated at the central and portal veins of Gp2 (Figure 9b).

## 4. Discussion

Retrospective studies showed that DM might be a significant cause of HCC. This may be due to the excessive production of reactive oxygen species (ROS) that alter the gene structures causing gene mutations which in turn led to the creation of undesired proteins [44]. Additionally, the previous studies on human showed the impact of metformin (MET) in decreasing the incidence of hepatocellular carcinoma in diabetic patients [12]. The current study was conducted to manifest a pre-diabetic rat’s model, which was then stimulated by diethylnitrosamine (DEN) for HCC. The study also aimed to explore the role of MET treatment in the pre-diabetic rats that were produced with DEN. Different treatments with MET were applied before and/or after induction with HCC.

As we have mentioned in the results section, the injection with STZ and DEN decreased body-weight when resembling their control untreated group. This reduction in body weight could be due to the toxic effect of STZ and DEN on the rats. Treatment with MET before and after induction with DEN marginally increased the body-weight, whichcould be due to the protective and therapeutic role of MET. This result was in covenant with preceding studies showing that MET improved the functions of different organs and enhanced the body weight in the pre-diabetic rats [45].

Induction of diabetes with STZ, followed by installation with DEN to form HCC, increased the ALT and AST which indicated the dysfunction of the liver. Also, cholesterol, triglycerides, and LDL increased in this group of rats. Furthermore, SOD, CAT, and GSH were decreased while MDA was raised in rats of (Gp2) when matched to their control. Such alterations in the liver, lipids profile, and oxidative status confirmed the damage of different organs after administration of both STZ and DEN. The carcinogen-DNA and oxidative DNA are adducts generated through carcinogen’s activities proposed an interactive role for ROS in the initiation stage. Thus, ROS has several impacts on the beginning of carcinogenesis by intermediating carcinogen activation, leading to the DNA injury and interrupting the repair of DNA. The final step of tumorigenesis (progression) comprises the irreversibility of the cancer growth from the pre-neoplastic cells of lesions [46]. The findings of the current study agree with previous studies that recorded liver damage post-injection with STZ [47,48], with DEN [49], or with both [50]. Since P450 enzyme’s activity is related to the generation of ROS, then the oxidative stress may have an essential role in the clonal amplification of these stimulatory cells. So, higher production of ROS has been found in neoplastic nodules in the liver of rats compared to the surrounding normal cells of the liver’s tissues [51]. Additionally, the oxidation of GSH by y-glutamyl transpeptidase in pre-neoplastic foci results in the formation of ROS [51]. Moreover, ROS may come from inflammatory cells as an extracellular origin [52].

Treatment with MET post-HCC induction in the diabetic rats ameliorated the damage caused by STZ and DEN. Furthermore, the treatment with MET post-HCC induction also improved the damage caused by both STZ and DEN. Our data came in convention with the pre-studies that revealed the possible role of MET in enhancing the damage on the liver in diabetic rats [50]. Treatment with MET pre- and post-induction of the pre-existing diabetes showed the maximum protection on the liver tissues as shown in the results section. This finding indicates that the treatment with MET either in diabetic alone or in diabetic/HCC is vital to decrease the severity of such diseases.

The histopathological changes in the tissues of the liver were consistent with the hematological and biochemical results. The STZ treated group that received STZ for 45 days followed by DEN for 60 days displayed marked histopathological alterations in the liver tissues. In the present experimental study, liver cirrhosis accompanied by the presence of many populations of lymphocytes and inflammatory cells was observed. On the contrary, MET treated groups displayed less dramatic histopathological changes, especially Gp5, in which the diabetic animals were treated with pre and post MET doses. Diethylnitrosamine has been recorded as a well-known hepatotoxin and hepatocarcinogen. The current study recorded an elevation of the liver enzymes (ALP and Arg) in serum, which is clear evidence for hepatocellular damage. This result is in agreeance with other researchers [53,54,55]. The liver enzymes are the most important indicators for diagnosis of the liver injury because they are located in the cytoplasm of the hepatocytes and they are released into the circulated blood after the incidence of the cellular injury [56,57].

Additionally, GSH has an essential role in many cellular homeostases, as the detoxification of endogenous and exogenous substances. Moreover, DEN is an electrophilic carcinogen that may interact with the sizeable nucleophilic pool of GSH, reducing the macromolecules and carcinogen interaction [58]. The present study revealed that GSH recorded a decrease in the DEN treated-group. So, many histopathological changes result in the STZ/DEN treated group due to the decline in GSH.

On the other hand, GSH elevated in the MET (both pre and post) treated groups, and there was a significant increase of GSH in the liver when matching with DEN-treated groups that came harmoniously with the idea of alleviation of DNA carcinogen interaction, thereby preventing a suitable environment for cancer-induction. Cirrhosis is one of the most common types of liver injury, which can cause HCC. Sufficient exposure to DEN could create an animal model, which looks like human HCC. Jo et al. (2016) [59] revealed a significant decrease in the level of p-AMPKα1 in the liver of DEN-treated rats, compared with control rats or those treated with MET. At low energy, AMPK (5′ adenosine monophosphate-activated protein kinase) enzyme plays an essential role in cellular energy homeostasis, to activate glucose and fatty acid uptake and oxidation. In response, AMPK activation stimulates the hepatic fatty acid oxidation, ketogenesis, glucose uptake, inhibition of cholesterol synthesis, lipogenesis, and triglyceride synthesis [60]. Our study showed that the biochemical analysis demonstrated an increase in cholesterol and triglyceride that was recorded in the STZ/DEN treated-group. The impact of MET on tumor growth including cellular studies are possibly explained by several mechanisms that are recorded in several studies [61,62,63,64].

Interestingly, numerous studies reported that MET has antiproliferative actions on untransformed epithelial cells via AMP-kinase dependent pathways, due to the role of AMP-kinase as an energy sensor that down-regulates processes, such as protein synthesis, when energy is in short supply. Additionally, there is an indirect mechanism reported that might be of considerable importance in subjects with high insulin levels and cancers with high levels of insulin and hybrid insulin/IGF-1 receptors [65,66].

Furthermore, increasing of the leukocytes that were assessed biochemically and confirmed histologically by apparent cellular infiltration were primarily diminished by pre/or post or pre/and post MET administration. According to these visible results, it can be concluded that MET ameliorates DEN-induced nephric injury through inhibitory apoptosis and interstitial inflammation.

In fact, it was found that AMPK is the principal target of MET, which acts as the sensor of cellular energy supplies and controls protein synthesis, apoptosis, and autophagy [67,68]. In most studies, MET has been reported to be associated with antineoplastic activity and decrease load of many types of tumors [69,70,71].

## 5. Conclusions

Taken together, the results of the present study highlight the protective role of MET when used as a treatment against DM or HCC. MET holds great promise for the improvement of the histology of hepatic tissue and liver function. Our findings offer important clinical implications for the treatment of DM and HCC, suggesting a role for MET as a novel therapeutic option to target the oxidative stress induced in DM and HCC. Further research is needed to endorse our finding, via studying at the molecular level the signaling pathways involved in the development of tumorigenesis in DM patients by different methods. This future work is predicted to give deep insight into the main molecular mechanism responsible for the bad prognosis of HCC in diabetic patients and the effect of MET in the improvement of such cases.

## Figures and Tables

**Table 1:** Hematological parameters of the groups of rats under study.

Groups	Hb (g/dL)	Hct (%)	Platelets (×10^3^/µL)	RBCs (×10^6^/µL)	WBCs (×10^3^/µL)
Negative control	12.5 ± 2.36	39.3 ± 6.19	623.2 ± 168.9 ^b^	5.95 ± 1.54 ^b^	10.3 ± 1.34 ^b^
STZ/DEN	14.64 ± 0.93	43.7 ± 3.16	584.6 ± 122.6 ^b^	9.35 ± 0.78 ^a^	17.02 ± 2.89 ^a^
STZ /MET/DEN	14.12 ± 2.75	46.2 ± 10.8	640.8 ± 78.21 ^b^	10.11 ± 1.65 ^a^	10.08 ± 1.41 ^b^
STZ/DEN/MET	15.82 ± 1.34	44.16 ± 3.13	1718.8± 285 ^a^	8.52 ± 0.49 ^a,b^	15.6 ± 2.81 ^a,b^
STZ/MET/DEN/MET	13.78 ± 1.06	42.02 ± 2.97	793 ± 70.29 ^b^	7.9 ± 0.89 ^a,b^	14.72 ± 2.50 ^a,b^
F-Value	1.31	0.55	25.45	7.60	5.73
*p*-Value	0.332 n.s.	0.707 n.s.	0.000	0.004	0.012

Hb: hemoglobin; Hct: hematocrit; RBCs: red blood cells; WBCs: white blood cells. Means that do not share a letter are significantly different. Different lower-case (a, b) letters indicate a significant difference between all the studied groups at *p* < 0.05 (Tukey’s test). n.s.: not significant.

**Table 2:** Serum alanine transaminase (ALT), aspartate transaminase (AST), total bilirubin (TB), total protein (TP), and albumin (Alb.) in the groups under study.

Groups	ALT (U/L)	AST (U/L)	TB (mg/dL)	TP (g/dL)	Alb. (g/dL)
Negative control	60 ± 5.5 ^d^	141 ± 8.5 ^d^	0.35 ± 0.04 ^b^	5.7 ± 0.35 ^a^	2.57 ± 0.28 ^a^
STZ/DEN	122.3 ± 8.7 ^a^	250.3 ± 11.2 ^a^	0.85 ± 0.07 ^a^	3.14 ± 0.48 ^c^	1.053 ± 0.2 ^c^
STZ/MET/DEN	92.3 ± 8.7 ^b,c^	191.3 ± 13.7 ^b,c^	0.47 ± 0.06 ^b^	4.15 ± 0.36 ^b,c^	1.8 ± 0.1 ^b^
STZ/DEN/MET	104.3 ± 8.7 ^a,b^	211.3 ± 10.03 ^b^	0.42 ± 0.05 ^b^	4.24 ± 0.44 ^b^	1.7 ± 0.29 ^b^
STZ/MET/DEN/MET	79.3 ± 7.8 ^c,d^	164.3 ± 11.1 ^c,d^	0.44 ± 0.053 ^b^	4.34 ± 0.32 ^b^	2 ± 0.25 ^a,b^
F-Value	26.57	43.95	37.90	16.05	16.33
*p*-Value	˂0.001	˂0.001	˂0.001	˂0.001	˂0.001

Different lower-case (a, b, c, d) letters indicate a significant difference between all the studied groups at *p* < 0.05 (Tukey’s test).

## Data Availability

The data presented in this study are available on request from the corresponding author.
